# Association between common polymorphisms in IL-1 and TNFα and risk of peri-implant disease: A meta-analysis

**DOI:** 10.1371/journal.pone.0258138

**Published:** 2021-10-05

**Authors:** Qiuchen Jin, Fangjun Teng, Zhigang Cheng

**Affiliations:** Department of Stomatology, The Central Hospital of Wuhan, Tongji Medical College, Huazhong University of Science and Technology, Wuhan, China; Khallikote University, INDIA

## Abstract

**Background:**

Pro-inflammatory cytokines interleukin-1 (IL-1) and tumor necrosis factor α (TNFα) play important roles in host immune response and bone metabolism during dental implant osseointegration. Whether the functional polymorphisms in IL-1α, IL-1β and TNFα were associated with peri-implant disease was unclear, and we performed the present meta-analysis for this purpose.

**Methods:**

Eligible studies investigating IL-1α C-889T, IL-1β C+3954T and C-511T, TNFα G-308A, composite genotype of IL-1α C-889T and IL-1β C+3954T for association with peri-implant disease, including peri-implantitis (PI), marginal bone loss (MBL) and implant failure/loss (IF/IL), were searched on several literature databases prior to April 30, 2021. Odds ratio (OR) and corresponding 95% confidence interval (CI) were calculated for each polymorphism in different genetic models and for composite genotype comparing carriers to non-carriers.

**Results:**

Twenty-seven studies (1324 cases with peri-implant disease and 1808 controls with healthy implants) were included. There was significant correlation between IL-1α C-889T and peri-implant disease in all genetic models. IL-1β C+3954T was associated with peri-implant disease risk in allelic (OR = 1.66, 95%CI 1.17–2.35, p = 0.004) and dominant model (OR = 1.74, 95%CI 1.19–2.53, p = 0.004), and in subgroups of Asians, Caucasians, non-smokers, IF/IL and PI. TT genotype of IL-1β C-511T increased the risk of peri-implant disease (OR = 1.68, 95%CI 1.15–2.43, p = 0.007) and MBL (OR = 4.33, 95%CI 1.72–10.9, p = 0.002) compared to CC+CT genotypes. We did not observed a significant association between TNFα G-308A and peri-implant diseases in overall or subgroups analysis. Carriers of positive composite genotype of IL-1α C-889T and IL-1β C+3954T had 1.95-fold (95%CI 1.35–2.80, p<0.001) risk of peri-implant disease and 1.76-fold (95%CI 1.05–2.95, p = 0.032) risk of IF/IL than non-carriers.

**Conclusion:**

Functional polymorphisms of IL-1α (C-889T), IL-1β (C+3954T, C-511T) and composite genotype of IL-1 can be used as predictive markers for peri-implant disease, whereas TNFα G-308A polymorphism was not associated with peri-implant disease.

## Introduction

Dental implants have been widely used as an effective treatment for edentulous patients with an increasing success rate and 10-year survival rate of dental implants in recent years [1,2]. However, implant failure still occurs in around 1.9~3.6% of dental-implant subjects [3]. Multiple factors, such as peri-implant complications, smoking behavior, implant technique and material, systemic medical condition of patients, play important roles in implant failure [4–6]. Among these factors, peri-implant disease, including peri-implantitis, marginal bone loss and implant loosening, are common biological complications in implant failure [7]. Peri-implantitis is triggered by bacterium infection and found in almost one-third of patients and one-fifth of all implants after 2-year follow-up [8]. The inflammatory process disrupts the function of soft tissue and supporting bone tissue around osseointegrated oral implants, results in progressive bone loss, and finally causes implant loss or implant failure [9].

A clusterization phenomenon has been observed that over half of the overall implant failures occurred in only one-third of all patients [10], indicating an individual susceptibility to implant failure. The endogenous predisposition may be mediated by host innate immune response to bacterium infection and implants, which subsequently influences individual’s susceptibility to peri-implant disease [11].

Implants may stimulate macrophages to release interleukin-1 (IL-1) and tumor necrosis factor α (TNFα) which are known strong pro-inflammatory cytokines [12]. In physiological situations, moderate expression of IL-1 and TNFα is required for the maintenance of low-grade inflammation and normal implant osseointegration [13]. However, excessive production of these mediators may trigger stronger inflammation response, disrupt the balance of bone resorption required for dental implant osseointegration, and finally elevate the risk of peri-implantitis and implant failure [13]. This is supported by the findings that significantly higher levels of IL-1β and TNFα in the peri-implant crevicular fluid (PICF) are observed in patients with peri-implantitis than in healthy controls [14]. Thus, IL-1 and TNFα, two crucial pro-inflammatory cytokines mediating the inflammation process of peri-implantitis, are potential predictive markers for development of peri-implant disease.

The expression of IL-1 and TNFα may be regulated by several functionally relevant polymorphisms, including IL-1α C-889T (rs1800587), IL-1β C+3954T (formally C+3953T, rs1143634) and C-511T (rs16944), and TNFα G-308A (rs1800629). The mutant alleles of these polymorphisms were found to increase the transcriptional activity of corresponding genes, resulting in overexpression of pro-inflammatory cytokines [11]. For example, a 1.23-fold increase in transcriptional activity of IL-1α rs1800587 TT genotype was found over the CC genotype [15]. The serum level of IL-1β was significantly higher in carriers of rs16944 TT genotype than those of CC genotype [16]. The -308A allele transcript of TNFα had 2-fold greater level of transcriptional activity than -308G transcript [17]. In total, these polymorphisms may modify the expression and production of IL-1 and TNFα, affect host immune response and susceptibility to inflammatory diseases. Previous studies demonstrated that TNFα G-308A [18,19], IL-1α C-889T [20] and IL-1β C+3954T [21] polymorphisms were associated with risk of chronic periodontitis. The association between these polymorphisms with susceptibility to implant failure, marginal bone loss and peri-implantitis have also been widely explored, which, however, yielded inconsistent results [22–24]. Most of these studies had a relatively small sample size and thus had insufficient statistic power to detect the genetic associations. Therefore, we performed a systematic review and meta-analysis, by quantitively synthesizing previous studies, for the association of common functional polymorphisms in IL-1 and TNFα with susceptibility to peri-implant disease.

## Methods

### Literature search strategy

We performed the present meta-analysis in accordance with Preferred Reporting Items for Systematic Reviews and Meta-Analysis (PRISMA 2020) statement [25] (S1 Table). Literature databases including PubMed, EMBASE, Web of Science, Google Scholar, Chinese National Knowledge Infrastructure (CNKI), Wanfang and Chinese Biomedical Literature Database (CBM) were searched for candidate studies related to our research topic from inception to April 30, 2021. Since literature search in Google scholar yielded a large number of unrelated articles, we only identified the first 200 records, which were sorted by relevance, for eligibility for meta-analysis. The following terms and their combinations were used: “dental implant”, “implant loss”, “peri-implant bone loss”, “peri-implantitis”, “peri-implant disease”, “marginal bone loss”, “MBL” AND “interleukin”, “tumor necrosis factor”, “IL-1”, “TNF” AND “polymorphism”, “variant”, “SNP”, “variation”. The detailed search strategy and search result for each database were listed in S2 Table. Additional candidate studies were obtained by manually reviewing the reference list of eligible studies included in the meta-analysis.

### Inclusion and exclusion criteria

Eligible studies should comply with these criteria: (1) Patients had one of the following peri-implant disease, including implant failure/loss (IF/IL), marginal bone loss (MBL) and peri-implantitis (PI), after dental implants while controls had successful or healthy implants; (2) Polymorphisms in IL-1α (C-889T, rs1800587), IL-1β (C+3954T, rs1143634; C-511T, rs16944), TNFα (G-308A, rs1800629), and composite IL-1 genotype (variant allele at both IL-1α C-889T and IL-1β C+3954T sites) were studied; (3) Genotype and/or allele distributions of polymorphisms in both groups were provided. Reviews, case reports and studies without sufficient genotype data for meta-analysis were excluded. For studies with duplicated samples, the most recent one was included. Since the controls were those who also had dental implants due to various dental diseases, they were not healthy controls representing the general population. Thus, Hardy-Weinberg Equilibrium (HWE) was not required for the control group.

### Quality assessment

We assessed the quality of included studies by using Newcastle-Ottawa Scale (NOS) for case-control study. Eight items with regard to “selection”, “comparability” and “exposure” categories were evaluated and a total of 9 stars were assigned. Any study with 7 or more stars according to NOS was considered as high-quality study.

### Data extraction

Two independent researchers extracted the following information of each study: first author, publication year, country, ethnicity, peri-implant condition, mean age, percent of smokers, genotyping method, sample size, genotype and allele distributions in both groups. Discrepancies, if occurred, were resolved by a third researcher.

### Statistical analysis

The meta-analysis was performed by using STATA 14.0 (StataCorp, TX, US). Between-study heterogeneity was assessed by I^2^ and Q test. I^2^ <50% and p value for Q test >0.10 indicated no obvious heterogeneity, and a fixed-effect model was used for quantitative synthesis analysis. Otherwise, a random-effect model was used since there was substantial heterogeneity. For each polymorphism, the odds ratio (OR) and 95% confidence interval (95%CI) were calculated to assess its association strength with risk of peri-implant disease in four comparison models: allelic (variant allele vs. wildtype allele), dominant (homozygous + heterozygous variant vs. homozygous wildtype), recessive (homozygous variant vs. homozygous + heterozygous wildtype) and homozygote model (homozygous variant vs. homozygous wildtype). For the composite genotype of IL-1α C-889T and IL-1β C+3954T, the OR for carriers vs. non-carriers were calculated. Subgroup analyses stratified by ethnicity (Asians, Caucasians), smoking status (non-smokers), peri-implant conditions (IF/IL, MBL, PI) were performed. Sensitivity analysis was also conducted by omitting one study at a time followed by pooling together the others. Publication bias was assessed by viewing the symmetry of funnel plot and Egger’s test. A p value <0.05 was considered as statistical significance.

## Results

### Description of eligible studies

**Fig 1:**
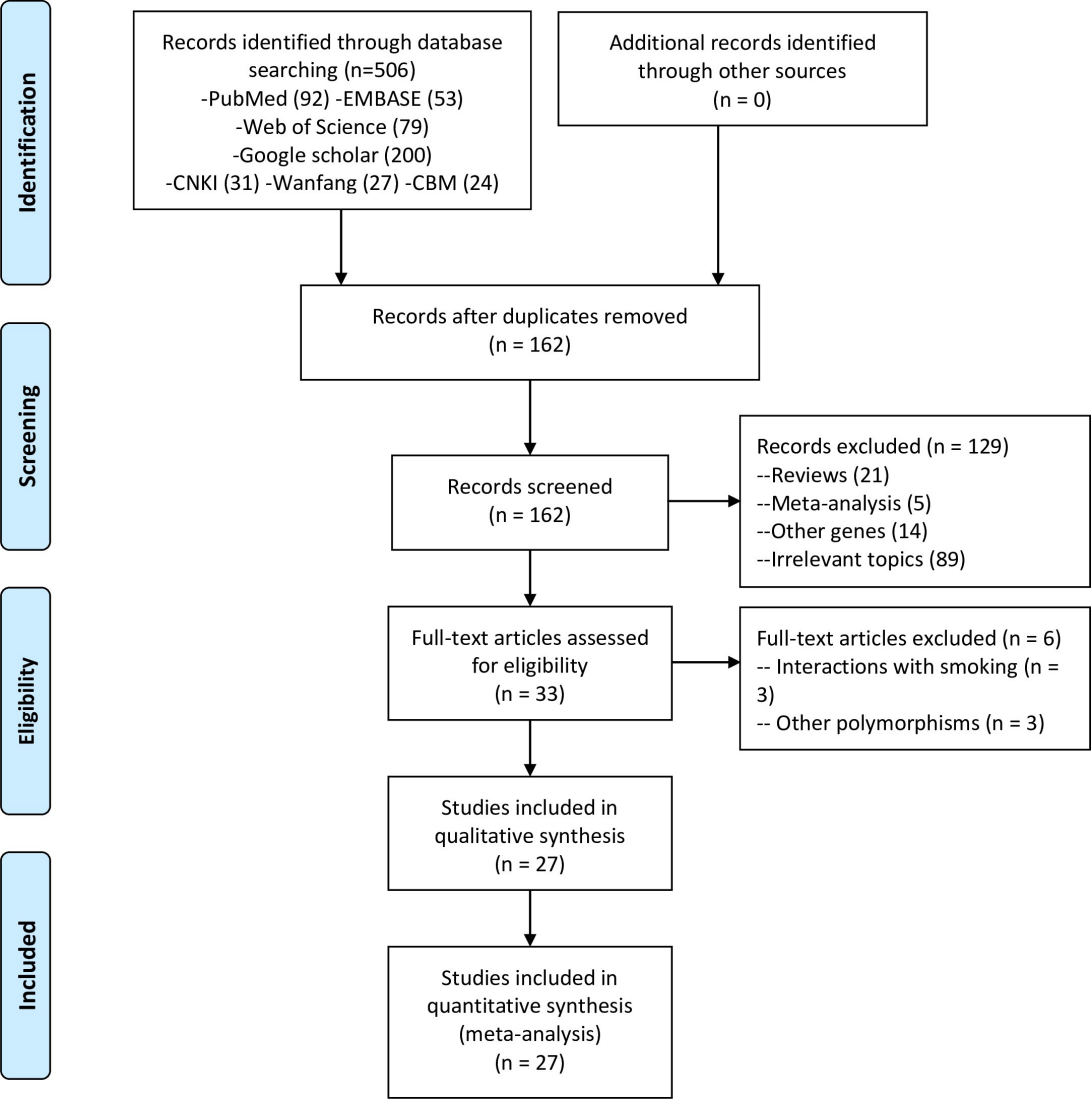
Flowchart of literature search.

As shown by the flow diagram of literature search (Fig 1), a total of 27 studies with 1324 cases and 1808 controls were finally included in meta-analysis [22–24,26–49]. There were 10, 3 and 13 studies recruiting patients with IF/IL, MBL and PI, respectively, while Fernandes *et al* [29] enrolled participants with various peri-implant disease. Six studies included participants of Asian ancestry while the others included subjects of Caucasian ancestry. Three studies were published in Chinese language [47–49] and the others were in English. The sample size ranged from 28 to 369. As to smoking status, 3 studies reported higher smoking rates in peri-implantitis group than healthy implant group [28,43,47], 7 enrolled participants matched for smoking status [27,32,33,36,39,40,45], 9 were performed in non-smokers [24,26,31,35,37,38,41,44,48] and 1 in smokers [30], whereas 7 did not report the smoking status [22,23,29,34,42,46,49]. IL-1α C-889T polymorphism was investigated in 10 studies, IL-1β C+3954T in 14 studies, IL-1β C-511T in 8 studies, TNFα G-308A in 12 studies, and composite genotype of IL-1α C-889T and IL-1β C+3954T in 7 studies. All studies were of high quality as they were awarded with 7 or more stars according to NOS. The characteristics of all eligible studies were summarized in Table 1, and the genotype data of each polymorphism was listed in S3–S7 Tables.

**Table 1:** Characteristics of all studies included in the meta-analysis.

Author (Year)	Country	Peri-implant condition	Sample size	Mean age, year	Percent of smokers, %	Genotyping method	IL-1α C-899T	IL-1β C+3954T	IL-1β C-511T	TNFα G-308A	Com^#^	NOS
Rogers (2002) [46]	Australia	Implant failure	19/31	66	NR	PCR-RFLP	Y	Y	N	N	Y	7
Shimpuku (2003) [45]	Japan	Marginal bone loss	17/22	55.2	36	PCR-RFLP	Y	Y	Y	N	N	7
Campos (2004) [44]	Brazil	Implant failure	28/38	47.2	0	PCR-RFLP	N	N	N	Y	N	8
Campos (2005) [24]	Brazil	Implant failure	28/34	47.5	0	PCR-RFLP	Y	Y	Y	N	Y	7
Laine (2006) [43]	Sweden	Peri-implantitis	71/49	67	67	PCR-RFLP	Y	Y	Y	N	Y	8
Lachmann (2007) [42]	German	Peri-implantitis	11/18	66	NR	NR	N	N	N	N	Y	8
Cury (2007) [41]	Brazil	Peri-implantitis	17/19	46	0	PCR-RFLP	N	N	N	Y	N	7
Lin (2007) [40]	China	Marginal bone loss	29/30	42.77	54	PCR-RFLP	Y	Y	Y	N	N	7
Montes (2009) [39]	Brazil	Implant loss	90/176	51.8	22	PCR-RFLP	N	Y	N	N	N	8
Cury (2009) [38]	Brazil	Peri-implantitis	49/41	48.4	0	PCR-RFLP	N	N	N	Y	N	8
Lu (2009) [47]	China	Marginal bone loss	18/26	47.8	52	PCR-RFLP	N	N	N	Y	N	8
Hamdy (2011) [37]	Egypt	Peri-implantitis	25/25	40.8	0	PCR-RFLP	N	N	N	N	Y	8
Dirschnabel (2011) [36]	Brazil	Implant loss	92/185	56.63	22	PCR-RFLP	N	N	Y	N	N	8
Gurol (2011) [34]	Turkey	Implant failure	16/23	NR	NR	ARMS-PCR	N	N	N	Y	N	7
Liu (2011) [48]	China	Peri-implantitis	50/50	46	0	PCR-RFLP	N	Y	Y	N	N	8
Melo (2012) [35]	Brazil	Peri-implantitis	16/31	45.98	0	PCR-RFLP	N	Y	Y	N	N	8
Vaz (2012) [23]	Portugal	Implant failure	55/100	NR	NR	TGP kits	N	N	N	N	Y	7
Jacobi-Gresser (2013) [33]	German	Implant failure	41/68	51.6	15	PCR-HRM	Y	Y	N	Y	N	7
Cosyn (2014) [32]	Belgium	Implant failure	14/14	65.5	21	Sequencing	Y	Y	Y	N	N	8
Rakic (2015) [31]	Serbia	Peri-implantitis	180/189	51.3	0	PCR-RFLP	N	N	N	Y	N	8
Garcia-Delanet (2015) [30]	Spain	Peri-implantitis	27/27	52.5	100	PST kits	Y	Y	N	N	N	8
Fernandes (2017) [29]	Portugal	Peri-implant disease	32/26	68.8	NR	PCR-RFLP	Y	Y	N	N	Y	7
Petkovic-Curcin (2017) [28]	Serbia	Peri-Implantitis	34/65	58	52	PCR-RFLP	N	N	N	Y	N	7
Li (2017) [49]	China	Peri-implantitis	90/95	NR	NR	PCR-RFLP	N	N	N	Y	N	8
Broker (2018) [27]	Brazil	Implant loss	81/163	51.6	19	Real-time PCR	N	N	N	Y	N	8
He (2020) [26]	China	Peri-implantitis	144/174	44.7	0	TaqMan	Y	Y	N	Y	N	8
Saremi (2021) [22]	Iran	Peri-implantitis	50/89	41	NR	PCR-RFLP	N	Y	N	Y	N	8

# Composite of IL-1B C+3954T and IL-1A C-899T polymorphisms; PCR: Polymerase chain reaction; RFLP: Restriction fragment length polymorphism; HRM: High resolution melting analysis; ARMS: Amplification refractory mutation system; NOS: Newcastle-Ottawa Scale; NR: Not reported.

### IL-1α C-889T polymorphism

**Fig 2:**
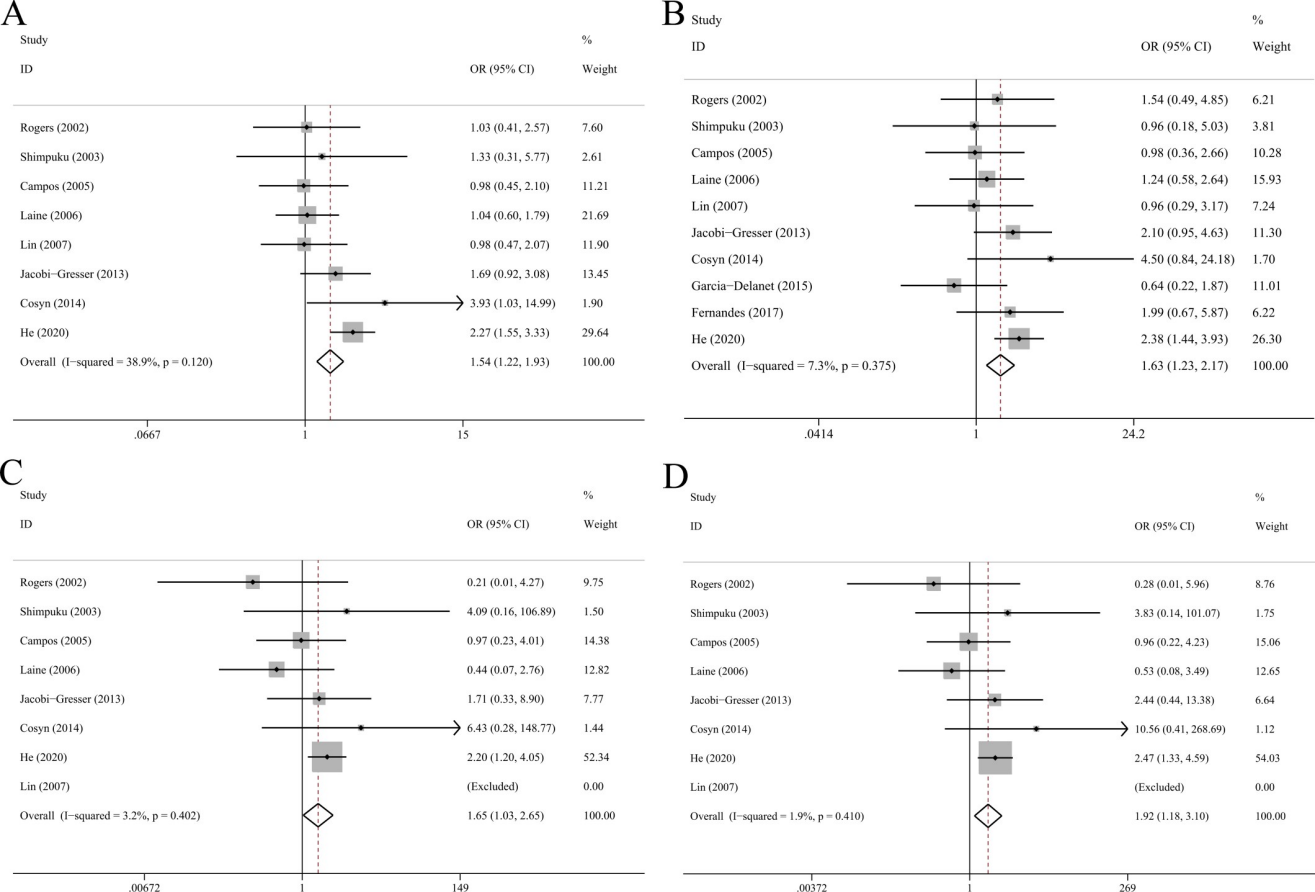
Forest plots of IL-1α C-889T polymorphism with peri-implant disease risk in allelic (A), dominant (B), recessive (C) and homozygote model (D).

The association between IL-1α C-889T and risk of peri-implant disease were investigated by 10 studies with 422 cases and 475 controls (Table 2). Meta-analysis using a fixed-effect model demonstrated that C-889T was associated with increased risk of peri-implant disease (T vs C: OR = 1.54, 95%CI 1.22–1.93, p<0.001; TT+CT vs CC: OR = 1.63, 95%CI 1.23–2.17, p = 0.001; TT vs CC+CT: OR = 1.65, 95%CI 1.03–2.65, p = 0.036; TT vs CC: OR = 1.92, 95%CI 1.18–3.10, p = 0.008; Fig 2). Carriers of TT or CT genotypes had higher risk of peri-implant disease in Asian populations (OR = 1.96, 95%CI 1.26–3.05, p = 0.003) and were more vulnerable to IF/IL (OR = 1.73, 95%CI 1.03–2.89, p = 0.037) compared with carriers of CC genotype.

**Table 2:** Association between IL-1α C-899T polymorphism and susceptibility to peri-implant disease.

Subgroup	Allele model, T vs C				Dominant model, TT+CT vs CC				Recessive model, TT vs CC+CT				Homozygote model, TT vs CC			
	No	OR (95%CI)	p	I^2^ (%)	No	OR (95%CI)	p	I^2^ (%)	No	OR (95%CI)	p	I^2^ (%)	No	OR (95%CI)	p	I^2^ (%)
Overall	8	1.54 (1.22–1.93)	<0.001	38.9	10	1.63 (1.23–2.17)	0.001	7.3	7	1.65 (1.03–2.65)	0.036	3.2	7	1.92 (1.18–3.10)	0.008	1.9
Ethnicity																
Asian	3	1.59 (0.85–2.94)	0.138	50.7	3	1.96 (1.26–3.05)	0.003	25.0	2	2.25 (1.24–4.10)	0.008	0	2	2.47 (1.33–4.59)	0.003	0
Caucasian	5	1.28 (0.93–1.76)	0.134	16.0	7	1.44 (1.00–2.08)	0.053	0	5	0.96 (0.43–2.12)	0.915	0	5	1.17 (0.51–2.65)	0.715	2.0
Non-smokers	2	1.59 (0.70–3.60)	0.264	73.1	2	1.70 (0.73–3.98)	0.220	59.0	2	1.93 (1.11–3.37)	0.020	7.9	2	2.14 (1.22–3.77)	0.008	24.9
Condition																
IF/IL	4	1.43 (0.96–2.13)	0.077	23.5	4	1.73 (1.03–2.89)	0.037	0	4	1.16 (0.47–2.81)	0.751	0	4	1.42 (0.57–3.55)	0.453	6.7
MBL	2	1.05 (0.54–2.03)	0.894	0	2	0.96 (0.36–2.53)	0.933	0	1	-	-	-	1	-	-	-
PI	2	1.57 (0.73–3.38)	0.246	81.2	3	1.39 (0.67–2.87)	0.374	64.2	2	1.26 (0.28–5.61)	0.761	62.2	2	1.49 (0.36–6.17)	0.580	56.9

IF/IL: Implant failure/loss; MBL: Marginal bone loss; PI: Peri-implantitis; No: Number of studies; OR: Odds ratio.

### IL-1β C+3954T polymorphism

**Fig 3:**
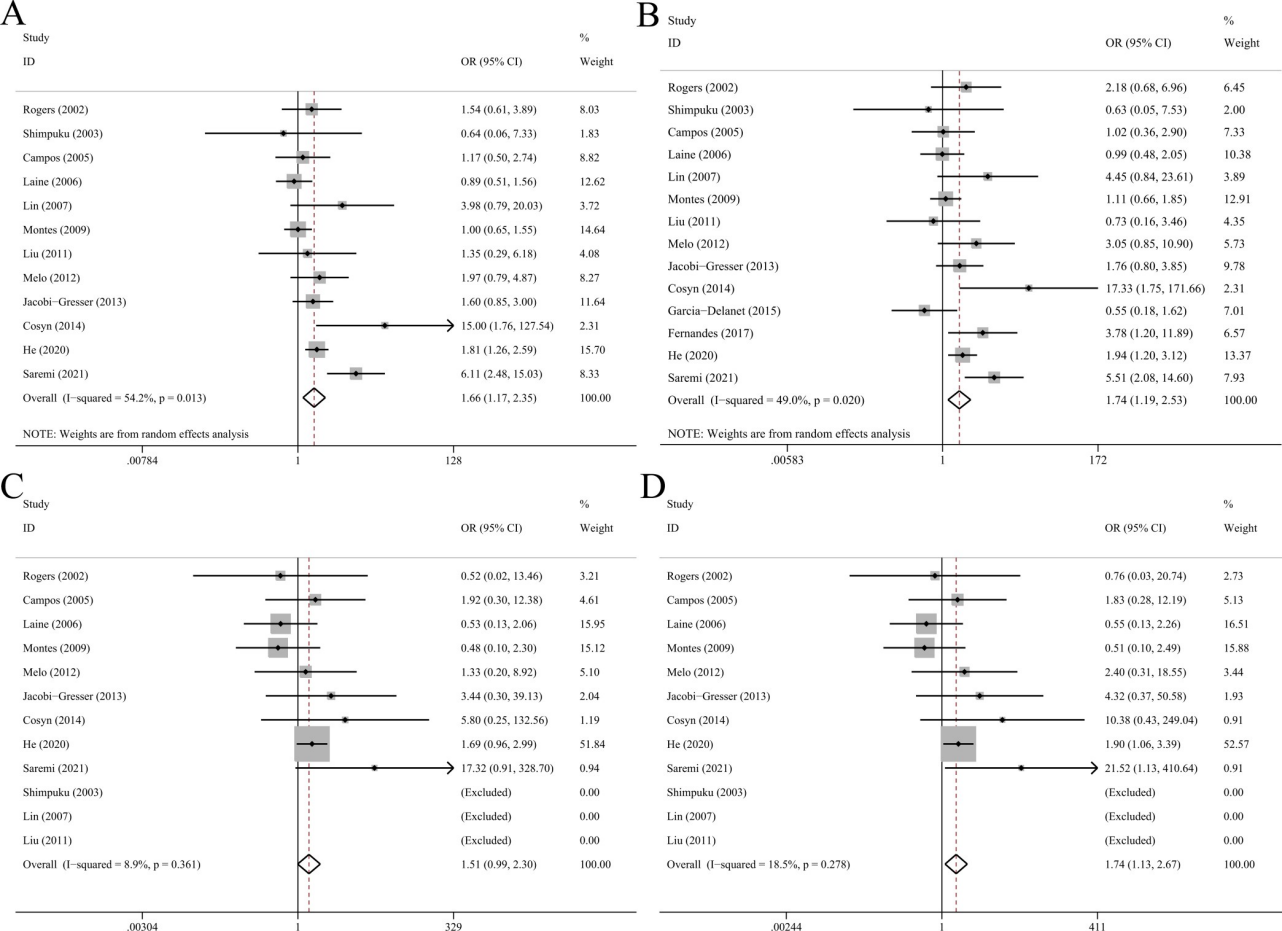
Forest plots of IL-1β C+3954T polymorphism with peri-implant disease risk in allelic (A), dominant (B), recessive (C) and homozygote model (D).

Fourteen eligible studies comprising 628 cases and 821 controls were incorporated (Table 3). Overall analysis showed significant correlation between IL-1β C+3954T and peri-implant disease (T vs C: OR = 1.66, 95%CI 1.17–2.35, p = 0.004, random-effect model; TT+CT vs CC: OR = 1.74, 95%CI 1.19–2.53, p = 0.004, random-effect model; TT vs CC: OR = 1.74, 95%CI 1.13–2.67, p = 0.012; Fig 3). Significant associations were also found in subgroups of Asians, Caucasians, non-smokers, IF/IL and PI.

**Table 3:** Association between IL-1β C+3954T polymorphism and susceptibility to peri-implant disease.

Subgroup	Allele model, T vs C				Dominant model, TT+CT vs CC				Recessive model, TT vs CC+CT				Homozygote model, TT vs CC			
	No	OR (95%CI)	p	I^2^ (%)	No	OR (95%CI)	p	I^2^ (%)	No	OR (95%CI)	p	I^2^ (%)	No	OR (95%CI)	p	I^2^ (%)
Overall	12	1.66 (1.17–2.35)	0.004	54.2	14	1.74 (1.19–2.53)	0.004	49.0	9	1.51 (0.99–2.30)	0.056	8.9	9	1.74 (1.13–2.67)	0.012	18.5
Ethnicity																
Asian	4	1.81 (1.29–2.54)	0.001	0	4	1.84 (1.20–2.82)	0.005	6.1	1	-	-	-	1	-	-	-
Caucasian	8	1.68 (1.05–2.68)	0.030	66.1	10	1.80 (1.11–2.90)	0.016	58.9	8	1.31 (0.70–2.46)	0.397	14.6	8	1.56 (0.83–2.96)	0.169	18.5
Non-smokers	4	1.70 (1.25–2.31)	0.001	0	4	1.73 (1.16–2.56)	0.007	4.1	3	1.68 (1.00–2.83)	0.052	0	3	1.92 (1.12–3.28)	0.017	0
Condition																
IF/IL	5	1.32 (0.98–1.78)	0.070	42.5	5	1.45 (1.01–2.09)	0.044	40.3	5	1.21 (0.51–2.88)	0.667	0	5	1.41 (0.59–3.35)	0.443	2.5
MBL	2	2.33 (0.67–8.05)	0.182	33.5	2	2.45 (0.69–8.74)	0.168	39.4	0	-	-	-	0	-	-	-
PI	5	1.87 (1.04–3.36)	0.038	69.5	6	1.59 (0.85–2.98)	0.149	64.6	4	1.61 (1.00–2.62)	0.052	41.7	4	1.86 (1.13–3.05)	0.014	46.2

IF/IL: Implant failure/loss; MBL: Marginal bone loss; PI: Peri-implantitis; No: Number of studies; OR: Odds ratio.

### IL-1β C-511T polymorphism

**Fig 4:**
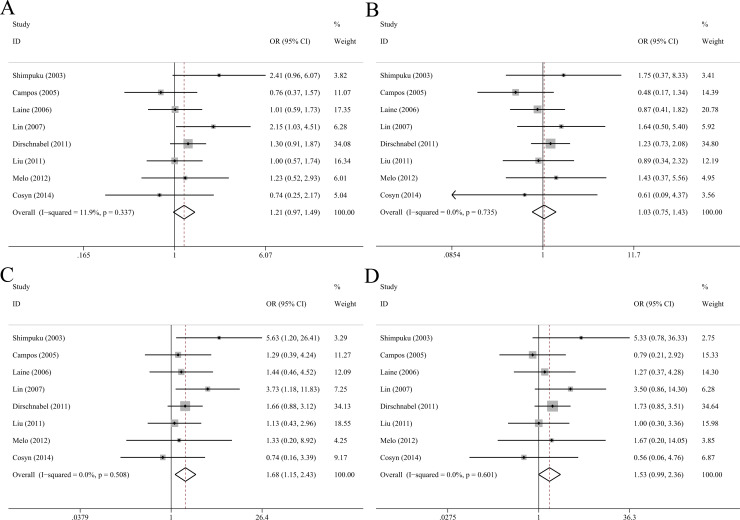
Forest plots of IL-1β C-511T polymorphism with peri-implant disease risk in allelic (A), dominant (B), recessive (C) and homozygote model (D).

Meta-analysis pooling 8 studies with 317 cases and 415 controls together showed that TT genotype of IL-1β C-511T polymorphism conferred significantly higher risk to peri-implant disease (TT vs CC+CT: OR = 1.53, 95%CI 1.15–2.43, p = 0.007) while T allele and TT+CT genotypes were not associated with disease risk (Table 4, Fig 4). Subgroup analysis suggested that IL-1β C-511T was associated with risk of MBL in allelic (OR = 2.25, 95%CI 1.27–4.01), recessive (OR = 4.33, 95%CI 1.72–10.9) and homozygote (OR = 4.06, 95%CI 1.31–12.6) models.

**Table 4:** Association between IL-1β C-511T polymorphism and susceptibility to peri-implant disease.

Subgroup	Allele model, T vs C				Dominant model, TT+CT vs CC				Recessive model, TT vs CC+CT				Homozygote model, TT vs CC			
	No	OR (95%CI)	p	I^2^ (%)	No	OR (95%CI)	p	I^2^ (%)	No	OR (95%CI)	p	I^2^ (%)	No	OR (95%CI)	p	I^2^ (%)
Overall	8	1.21 (0.97–1.49)	0.086	11.9	8	1.03 (0.75–1.43)	0.840	0	8	1.68 (1.15–2.43)	0.007	0	8	1.53 (0.99–2.36)	0.056	0
Ethnicity																
Asian	3	1.48 (1.00–2.19)	0.053	49.9	3	1.23 (0.63–2.40)	0.542	0	3	2.55 (0.95–6.83)	0.062	50.8	3	2.1 (0.94–4.70)	0.079	29.9
Caucasian	5	1.11 (0.86–1.43)	0.429	0	5	0.98 (0.68–1.42)	0.914	0	5	1.42 (0.90–2.26)	0.133	0	5	1.34 (0.79–2.24)	0.275	0
Non-smokers	3	0.96 (0.65–1.42)	0.847	0	3	0.79 (0.42–1.45)	0.429	0	3	1.21 (0.60–2.42)	0.599	0	3	0.98 (0.43–2.23)	0.961	0
Condition																
IF/IL	3	1.13 (0.83–1.53)	0.442	23.5	3	0.98 (0.63–1.54)	0.940	28.2	3	1.43 (0.84–2.41)	0.183	0	3	1.33 (0.73–2.41)	0.348	0
MBL	2	2.25 (1.27–4.01)	0.006	0	2	1.68 (0.65–4.39)	0.281	0	2	4.33 (1.72–10.9)	0.002	0	2	4.06 (1.31–12.6)	0.015	0
PI	3	1.04 (0.73–1.49)	0.832	0	3	0.95 (0.55–1.62)	0.839	0	3	1.26 (0.64–2.50)	0.505	0	3	1.19 (0.54–2.63)	0.673	0

IF/IL: Implant failure/loss; MBL: Marginal bone loss; PI: Peri-implantitis; No: Number of studies; OR: Odds ratio.

### TNFα G-308A polymorphism

**Fig 5:**
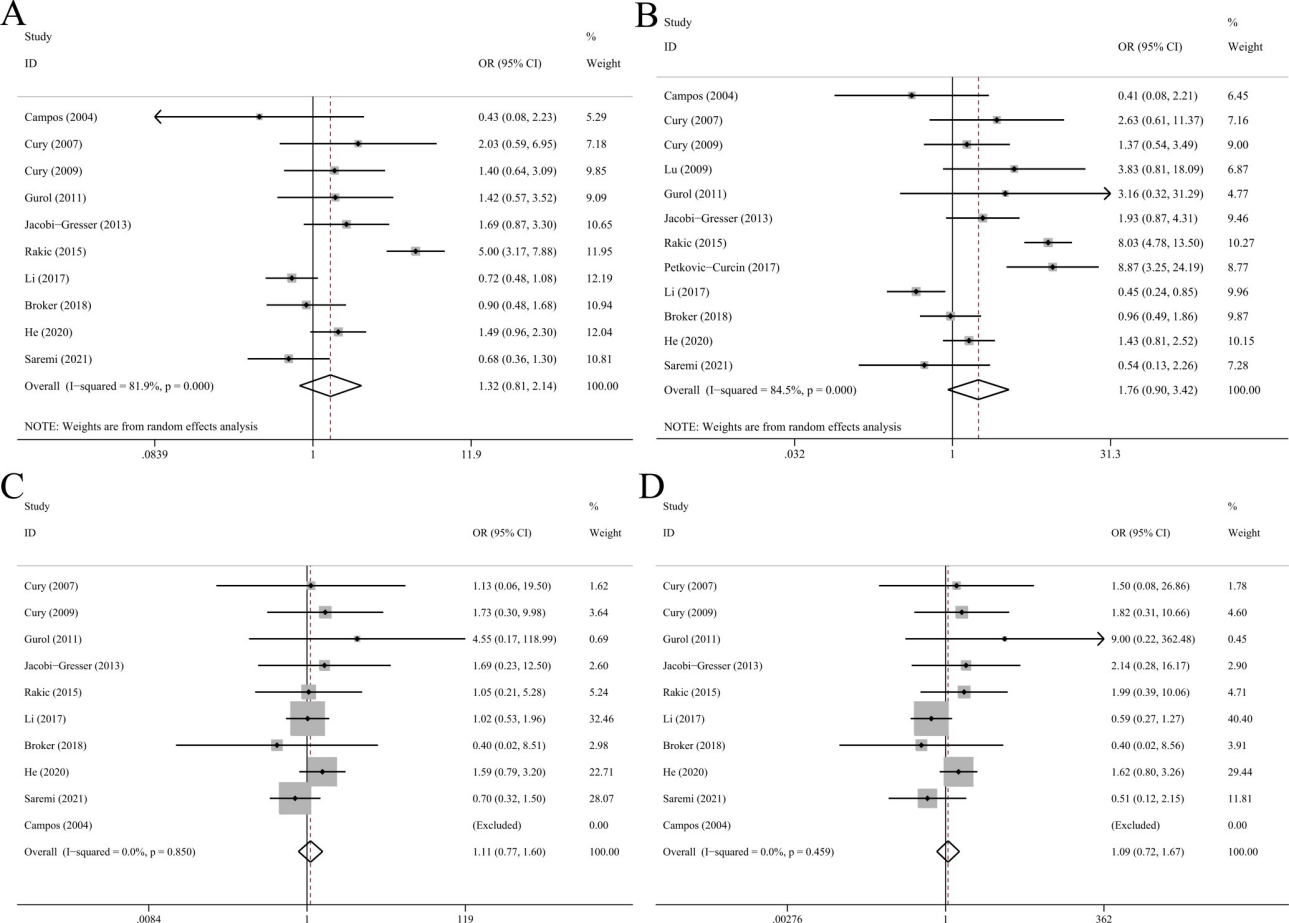
Forest plots of TNFα G-308A polymorphism with peri-implant disease risk in allelic (A), dominant (B), recessive (C) and homozygote model (D).

As shown in Table 5, overall analysis incorporating 12 eligible studies with 748 cases and 990 controls demonstrated that TNFα G-308A was not associated with risk of peri-implant disease in all comparison models (Fig 5). Moreover, there were no significant correlations in subgroup analyses stratified by ethnicity, smoking status and peri-implant conditions.

**Table 5:** Association between TNFα G-308A polymorphism and susceptibility to peri-implant disease.

Subgroup	Allele model, A vs G				Dominant model, AA+GA vs GG				Recessive model, AA vs GG+GA				Homozygote model, AA vs GG			
	No	OR (95%CI)	p	I^2^ (%)	No	OR (95%CI)	p	I^2^ (%)	No	OR (95%CI)	p	I^2^ (%)	No	OR (95%CI)	p	I^2^ (%)
Overall	10	1.32 (0.81–2.14)	0.267	81.9	12	1.76 (0.90–3.42)	0.098	84.5	9	1.11 (0.77–1.60)	0.569	0	9	1.09 (0.72–1.67)	0.672	0
Ethnicity																
Asian	2	1.03 (0.50–2.10)	0.928	82.4	3	1.15 (0.40–3.30)	0.793	80.7	2	1.25 (0.78–2.02)	0.350	0	2	0.99 (0.37–2.66)	0.978	72.4
Caucasian	8	1.42 (0.76–2.60)	0.271	80.8	9	2.02 (0.93–4.39)	0.076	81.8	7	0.94 (0.53–1.65)	0.819	0	7	1.27 (0.61–2.65)	0.527	0
Non-smokers	5	1.81 (0.86–3.81)	0.116	80.6	5	1.96 (0.70–5.45)	0.198	86.0	4	1.50 (0.83–2.70)	0.176	0	4	1.68 (0.93–3.03)	0.087	0
Condition																
IF/IL	4	1.16 (0.78–1.70)	0.464	12.7	4	1.20 (0.75–1.91)	0.439	26.0	3	1.39 (0.36–5.39)	0.631	0	3	1.63 (0.41–6.40)	0.484	0
MBL	0	-	-	-	1	-	-	-	0	-	-	-	0	-	-	-
PI	6	1.46 (0.72–2.96)	0.288	89.0	7	1.93 (0.73–5.13)	0.186	90.3	6	1.09 (0.75–1.59)	0.645	0	6	1.05 (0.68–1.64)	0.818	11.3

IF/IL: Implant failure/loss; MBL: Marginal bone loss; PI: Peri-implantitis; No: Number of studies; OR: Odds ratio.

### Composite genotype of IL-1α C-889T and IL-1β C+3954T

**Fig 6:**
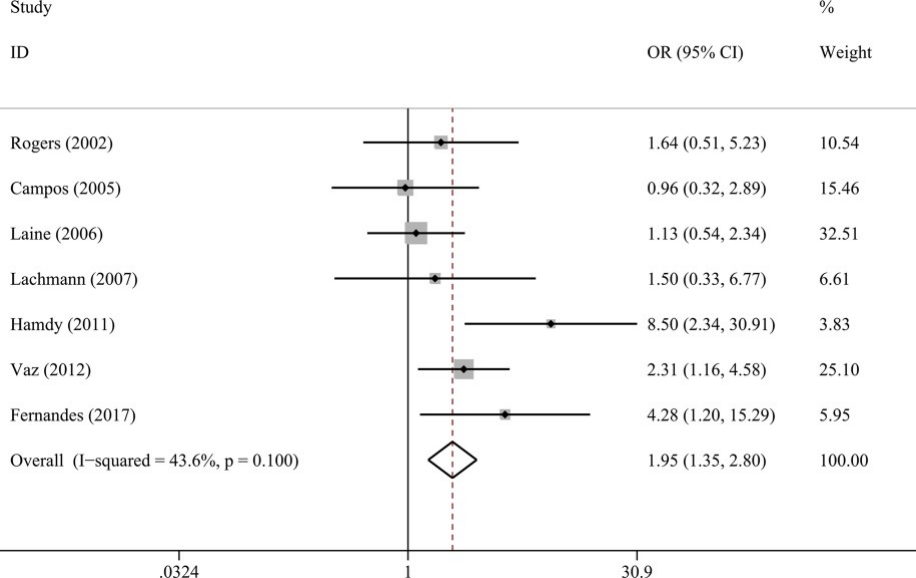
Forest plots of composite genotype of IL-1α C-889T and IL-1β C+3954T with peri-implant disease risk.

Seven studies comprising 241 cases and 283 controls were included, which were all of Caucasian ancestry. Carriers of composite genotype of IL-1α C-889T and IL-1β C+3954T had a higher risk of peri-implant disease (OR = 1.95, 95%CI 1.35–2.80, p<0.001, Fig 6) and IF/IL (OR = 1.76, 95%CI 1.05–2.95, p = 0.032, I^2^ = 0) than non-carriers using a fixed-effect model. The composite genotype was not associated with peri-implantitis (OR = 2.31, 95%CI 0.65–8.16, p = 0.195, I^2^ = 72.2%).

### Sensitivity analysis

Sensitivity analysis demonstrated that the pooled association of IL-1α C-889T with peri-implant disease was significantly affected by the study of He *et al* [26]. When we excluded this study, IL-1α C-889T was no longer associated with risk pf peri-implant disease in all comparison models (T vs C: OR = 1.23, 95%CI 0.92–1.64, p = 0.160; TT+CT vs CC: OR = 1.37, 95%CI 0.97–1.92, p = 0.075; TT vs CC+CT: OR = 1.06, 95%CI 0.49–2.26, p = 0.889; TT vs CC: OR = 1.27, 95%CI 0.58–2.78, p = 0.557) by using a fixed-effect model (I^2^ = 0).

### Publication bias

The funnel plots of all polymorphisms under various comparison models were symmetric, and p values of Egger’s test were all >0.05 (S8 Table), indicating that there was no evidence of obvious publication bias.

## Discussion

The present meta-analysis, by incorporating 3132 dental implant patients from 27 studies, demonstrated that functional polymorphisms in genes encoding pro-inflammatory IL-1α (C-889T, rs1800587), IL-1β (C+3954T, rs1143634; C-511T, rs16944) and the composite genotype of the polymorphic sites were associated with susceptibility to peri-implant disease. However, the meta-analysis did not reveal an association between the functional polymorphism in another pro-inflammatory gene TNFα (G-308A, rs1800629) and the disease risk.

IL-1 is a pivotal inflammatory cytokine mediating immune response and bone metabolism in dental implants [50]. It plays a crucial role in osseointegration process by stimulating the production of prostaglandins (e.g. prostaglandin E2) associated with enhanced bone resorption as well as the production of matrix metalloproteinases (MMPs) that augment collagen degradation [51]. IL-1α and IL-1β are the most studied members of IL-1. PICF level of IL-1β was significantly elevated in peri-implantitis sites than in healthy implant sites [52]. Functional experiments showed that T alleles of IL-1α -889 site, IL-1β +3954 and -511 sites increased the transcriptional activity of corresponding genes [11], suggesting that they were risk alleles of peri-implant disease. A previous meta-analysis pooling together 13 eligible studies found that IL-1α C-889T and IL-1β C+3954T were not associated with peri-implant disease [53]. On the contrary, the present meta-analysis, which included more recent studies and had the largest sample size, demonstrated that both polymorphisms were predictive markers for peri-implant disease. We also found positive associations of these polymorphisms with implant failure/loss and peri-implantitis. Moreover, our analysis yielded additional findings that TT genotype of IL-1β C-511T increased the risk of peri-implant disease and the polymorphism was a potential marker for marginal bone loss. Overall, the present meta-analysis provides new evidence for the predictive value of IL-1 gene functional polymorphisms in peri-implant diseases.

In addition to the genetic association of individual variant, we also investigated the composite genotype of IL-1α C-889T and IL-1β C+3954T variants. Patients with risk alleles at both IL-1α C-889T and IL-1β C+3954T sites had 1.95-fold risk of peri-implant disease and 1.76-fold risk of implant failure/loss than non-carriers. The effect size magnitude of composite genotype was larger than that of a single variant in dominant model.

Different from the mechanism of IL-1, TNFα stimulates bone resorption directly by promoting the differentiation and maturation of osteoclasts and enhancing their resorptive activity, or indirectly through interaction with receptor activation nuclear factor kappa-B (RANK) and its ligand (RANKL) [54]. The promoter G-308A polymorphism strongly modulates the transcriptional activity of TNF-α, of which the -308A allelic form has up to five-fold transcription level than the -308G form [16,55]. Increased production of TNF-α may trigger excessive osteoclastic bone resorption and promote the development of inflammatory bone diseases, such as rheumatoid arthritis and periodontal disease [54]. AA genotype of G-308A site was found to be associated with increased chronic and aggressive periodontitis risk [56]. Previous studies revealed that TNF-α level was significantly higher in PISF from peri-implantitis sites than that in healthy peri-implant tissue [57,58], indicating that TNF-α may be involved in the development of peri-implant disease. In two case-control studies, TNF-α G-308A polymorphism was associated with increased risk of peri-implantitis in dominant model (AA+GA vs GG), which was still significant after adjustment for smoking and positive history of periodontitis [28,31]. However, the other studies failed in finding positive relationship, and our meta-analysis revealed no significant association of G-308A with peri-implant disease or subgroups of IF/IL and peri-implantitis. The results of our meta-analysis incorporating more eligible studies was consistent with a previous one [59]. Whether TNF-α is absolutely required for osteoclastogenesis and bone resorption during dental implant osseointegration is still in debate [54]. The pathogenic role of TNF-α and predictive value of its functional polymorphisms for peri-implant disease need further investigation.

Smoking status is a well-known risk indicator for peri-implant disease [60]. Smoking and positive IL-1 genotype were found to have an synergistic effect resulting in more implant complications [61–63]. Thus, smoking status may be a confounding factor for the genetic associations. Only one study was performed in smokers, which showed no association between IL-1 polymorphisms and peri-implantitis [30]. We did not perform subgroup analysis of smokers due to insufficient data but conducted subgroup analysis in non-smokers for the first time. In non-smoking, dental implant patients, IL-1α C-899T and IL-1β C+3954T polymorphisms were both significantly associated with susceptibility to peri-implant disease, implying an independent role of these polymorphisms in the development of peri-implant disease.

Periodontitis is another risk factor of peri-implant disease [64]. The unbalanced distribution of periodontitis history between peri-implant disease and healthy implant groups may bias the genetic association. In three studies included in our meta-analysis, significantly higher rate of periodontitis history was found in patients with peri-implantitis compared to those with healthy implant [26,28,30]. Sensitivity analysis revealed that IL-1α C-889T was no longer associated with peri-implant disease risk after excluding the study of He *et al* [26].

Some limitations of our meta-analysis should be addressed. Firstly, our meta-analysis only provided overall estimates and subgroup analyses stratified by ethnicity and peri-disease condition, while the other confounding factors, such as history of periodontitis, smoking behavior and oral hygiene, were not taken into account because of unavailable data. Pooling analysis of patient-level data for confounding factors and genotypes will make it possible to explore the gene-environment interactions and shed light on the independent role of a single polymorphism in the development of peri-implant disease. Secondly, the term peri-implant disease is a heterogenous condition including several dental implant complications, each of which may have different pathogenic mechanism and is clinically defined under various inclusion/exclusion criteria between studies. This may contribute to the heterogeneity of our meta-analysis and the overall association estimates should be cautiously interpreted. Thirdly, the sample size was still relatively small, especially for analysis of IL-1α C-889T, IL-1β C-511T and composite genotype of IL-1α C-889T and IL-1β C+3954T. More large-scale, prospective cohort studies are warranted in the future.

In conclusion, the present meta-analysis with the largest sample size demonstrates that IL-1α C-889T, IL-1β C+3954T and C-511T, and composite genotype of IL-1α C-889T and IL-1β C+3954T, but not TNFα G-308A, are potential predictive markers of peri-implant disease.

## Supporting information

S1 Table PRISMA 2020 checklist. (DOCX)

S2 Table Detailed search strategy. (DOCX)

S3 Table Genotype data for IL-1α C-899T in association with peri-implant disease. (XLSX)

S4 Table Genotype data for IL-1β C+3954T in association with peri-implant disease. (XLSX)

S5 Table Genotype data for IL-1β C-511T in association with peri-implant disease. (XLSX)

S6 Table Genotype data for TNFα G-308A in association with peri-implant disease. (XLSX)

S7 Table Composite genotype data of IL-1α C-899T and IL-1β C+3954T. (XLSX)

S8 Table Results of Egger’s test. (DOCX)
