# Supplementary material for: Association between common polymorphisms in IL-1 and TNFα and risk of peri-implant disease: A meta-analysis
Source: PLoS One. 2021 Oct 5;16(10):e0258138. doi: 10.1371/journal.pone.0258138 (PMC8491952; doi:10.1371/journal.pone.0258138)
Supplement: S2 Table — (DOCX) [file pone.0258138.s002.docx]

S2 Table. Detailed search strategy.

| Databases | Search strategy | Results |
| --- | --- | --- |
| PubMed | (“dental implant”[tiab] OR “implant loss”[tiab] OR “implant failure”[tiab] OR “peri-implant bone loss”[tiab] OR peri-implantitis[tiab] OR “peri-implant disease”[tiab] OR “marginal bone loss”[tiab] OR MBL[tiab]) AND (interleukin[tiab] OR “tumor necrosis factor”[tiab] OR IL-1[tiab] OR TNF[tiab]) AND (polymorphism*[tiab] OR variant*[tiab] OR SNP[tiab] OR variation*[tiab]) | 92 |
| EMBASE | (“dental implant” OR “implant loss” OR “implant failure” OR “peri-implant bone loss” OR peri-implantitis OR “peri-implant disease” OR “marginal bone loss” OR MBL) AND (interleukin OR “tumor necrosis factor” OR IL-1 OR TNF) AND (polymorphism OR variant OR SNP OR variation) | 53 |
| Web of Science | (“dental implant” OR “implant loss” OR “implant failure” OR “peri-implant bone loss” OR peri-implantitis OR “peri-implant disease” OR “marginal bone loss” OR MBL) AND (interleukin OR “tumor necrosis factor” OR IL-1 OR TNF) AND (polymorphism OR variant OR SNP OR variation) | 79 |
| Google scholar | (“dental implant” OR “implant loss” OR “implant failure” OR “peri-implant bone loss” OR peri-implantitis OR “peri-implant disease” OR “marginal bone loss” OR MBL) AND (interleukin OR “tumor necrosis factor” OR IL-1 OR TNF) AND (polymorphism OR variant OR SNP OR variation) | First 200 |
| CNKI | (种植体 OR 边缘骨吸收) AND (多态性) | 31 |
| Wangfang | (种植体 OR 边缘骨吸收) AND (多态性) | 27 |
| CBM | (种植体 OR 边缘骨吸收) AND (多态性) | 24 |
