# Supplementary material for: Association between common polymorphisms in IL-1 and TNFα and risk of peri-implant disease: A meta-analysis
Source: PLoS One. 2021 Oct 5;16(10):e0258138. doi: 10.1371/journal.pone.0258138 (PMC8491952; doi:10.1371/journal.pone.0258138)
Supplement: S8 Table — (DOCX) [file pone.0258138.s008.docx]

S8 Table. Results of Egger’s test.

| Polymorphism | Coefficient | Standard error | t | p |
| --- | --- | --- | --- | --- |
| IL-1α C-889T rs1800587 |  |  |  |  |
| Allele model, T vs C | -0.934 | 1.209 | -0.77 | 0.469 |
| Dominant model, TT+CT vs CC | -1.138 | 0.899 | -1.27 | 0.241 |
| Recessive model, TT vs CC+CT | -0.623 | 0.690 | -0.90 | 0.408 |
| Homozygote model, TT vs CC | -0.512 | 0.699 | -0.73 | 0.497 |
| IL-1βC+3954T rs1143634 |  |  |  |  |
| Allele model, T vs C | 1.004 | 0.856 | 1.17 | 0.268 |
| Dominant model, TT+CT vs CC | 0.776 | 0.868 | 0.89 | 0.389 |
| Recessive model, TT vs CC+CT | 0.160 | 0.655 | 0.24 | 0.815 |
| Homozygote model, TT vs CC | 0.323 | 0.684 | 0.47 | 0.651 |
| IL-1βC-511T rs16944 |  |  |  |  |
| Allele model, T vs C | 0.056 | 1.189 | 0.05 | 0.964 |
| Dominant model, TT+CT vs CC | -0.188 | 0.780 | -0.24 | 0.818 |
| Recessive model, TT vs CC+CT | 0.231 | 1.094 | 0.21 | 0.840 |
| Homozygote model, TT vs CC | -0.050 | 0.943 | -0.05 | 0.960 |
| TNFα G-308A rs1800629 |  |  |  |  |
| Allele model, A vs G | -0.857 | 2.183 | -0.39 | 0.705 |
| Dominant model, AA+GA vs GG | -0.661 | 1.900 | -0.35 | 0.735 |
| Recessive model, AA vs GG+GA | 0.250 | 0.476 | 0.52 | 0.616 |
| Homozygote model, AA vs GG | 0.460 | 0.671 | 0.69 | 0.515 |
| Composite genotype of IL-1α C-889T and IL-1β C+3954T | 1.422 | 1.838 | 0.77 | 0.474 |
